# Extracellular glucose triggers metabolic reprogramming of cultured human bronchial epithelial cells and indirect fibroblast activation

**DOI:** 10.1002/2211-5463.13852

**Published:** 2024-07-01

**Authors:** Sangmi S. Park, Rafael Ward, Patrick Geraghty, Itsaso Garcia‐Arcos

**Affiliations:** ^1^ Department of Cell Biology State University of New York Downstate Health Sciences University Brooklyn NY USA; ^2^ Department of Medicine State University of New York Downstate Health Sciences University Brooklyn NY USA

**Keywords:** epithelial cells, fibroblast activation, glucose, metabolic reprogramming

## Abstract

Glucose is essential for energy metabolism, and its usage can determine other cellular functions, depending on the cell type. In some pathological conditions, cells are exposed to high concentrations of glucose for extended periods. In this study, we investigated metabolic, oxidative stress, and cellular senescence pathways in human bronchial epithelial cells (HBECs) cultured in media with physiologically low (5 mm) and high (12.5 mm) glucose concentrations. HBECs exposed to 12.5 mm glucose showed increased glucose routing toward the pentose phosphate pathway, lactate synthesis, and glycogen, but not triglyceride synthesis. These metabolic shifts were not associated with changes in cell proliferation rates, oxidative stress, or cellular senescence pathways. Since hyperglycemia is associated with fibrosis in the lung, we asked whether HBECS could activate fibroblasts. Primary human lung fibroblasts cultured in media conditioned by 12.5 mm glucose‐exposed HBECs showed a 1.3‐fold increase in the gene expression of *COL1A1* and *COL1A2*, along with twofold increased protein levels of smooth muscle cell actin and 2.4‐fold of COL1A1. Consistently, HBECs cultured with 12.5 mm glucose secreted proteins associated with inflammation and fibrosis, such as interleukins IL‐1β, IL‐10, and IL‐13, CC chemokine ligands CCL2 and CCL24, and with extracellular matrix remodeling, such as metalloproteinases (MMP)‐1, MMP‐3, MMP‐9, and MMP‐13 and tissue inhibitors of MMPs (TIMP)‐1 and ‐2. This study shows that HBECs undergo metabolic reprogramming and increase the secretion of profibrotic mediators following exposure to high concentrations of glucose, and it contributes to the understanding of the metabolic crosstalk of neighboring cells in diabetes‐associated pulmonary fibrosis.

## Graphical abstract

Hyperglycemia is associated with lung fibrosis. We found that culturing human bronchial epithelial cells with high levels of glucose changed their metabolism, increasing the activity of the pentose phosphate pathway, lactate synthesis, and glycogen synthesis. High glucose also promoted the secretion of profibrotic mediators to the culture media, and fibroblasts cultured with this media became activated.

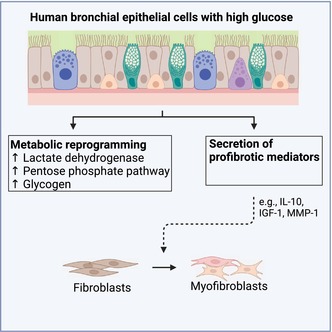

**Abbreviations**
ASL: airway surface liquid
BSA: bovine serum albumin
CCL: CC chemokine ligand
ECM: extracellular matrix
EMT: epithelial to mesenchymal transition
FBS: fetal bovine serum
G6P: glucose‐6‐phosphate
G6PDH: glucose‐6‐phosphate dehydrogenase
HBEC: bronchial epithelial cell
hFGF: human fibroblastic growth factor
HK: hexokinase
IGF: insulin growth factor
IL: interleukin
IPF: idiopathic pulmonary fibrosis
LDH: lactate dehydrogenase
MMP: metalloproteinase
NADPH: nicotinamide adenine dinucleotide phosphate
OCR: oxygen consumption rate
PBS: phosphate‐buffered saline
PCNA: proliferating cell nuclear antigen
PDH: pyruvate dehydrogenase
PDK: pyruvate dehydrogenase kinase
PPP: pentose phosphate pathway
ROS: reactive oxygen species
STZ: streptozotocin
T1D: type 1 diabetes
TBST: tris‐buffer saline with Tween 20
TCA: tricarboxylic acid cycle
TGF‐β: transforming growth factor beta
TIMP: tissue inhibitor of metalloproteinase
αSMA: alpha smooth muscle actin

The differential use of energy substrates by distinct cell types is a field that has recently received increasing interest, and metabolic reprogramming is described for fibroblasts and immune cells in multiple pathological conditions [1, 2]. However, little is known about the substrates used by epithelial cells, and specifically bronchial epithelial cells, despite their involvement in initiating steps of inflammatory cascades associated with lung disease [3].

In the lungs, the airway epithelium together with the capillary endothelium constitutes a barrier that separates the airway lumen from the internal environment. The luminal surface of the epithelium is covered by a thin layer of fluid called the airway surface liquid (ASL), which is frequently exposed to bacteria, fungi, and viruses present in the environment [4]. The glucose concentration in the ASL is actively maintained at approximately 0.4 mm, much lower than in plasma (5 mm) [5], to limit bacterial growth and prevent respiratory infections. The bronchial epithelium is polarized, with the differentiated apical side exposed to the air and the basal side exposed to the lung parenchyma. Bronchial epithelial cells can take up glucose from both basal and apical sides via GLUT transporters [6]. Glucose entering through the basal side would be of blood origin, while glucose entering through the apical side would be part of active clearance from the ASL.

Glucose concentration in ASL is increased during hyperglycemia [6]. Airway epithelial cells rapidly metabolize glucose when exposed to high concentrations by increasing glycolysis, glycogen, and sorbitol [7]. However, hyperglycemia can increase the intracellular generation of reactive oxygen species (ROS) [8], damage airway epithelial cells [9], and promote cellular senescence in animal and cell models [10, 11].

Type 1 diabetes (T1D), characterized by long‐term hyperglycemia, is an independent risk factor for pulmonary fibrosis [12]. Animal models of T1D show fibrotic modifications of the lung architecture [13]. We recently described increased airway subepithelial collagen deposition in diabetic mice [14]. Whether airway epithelial cells contribute to airway remodeling and fibroblast activation during pulmonary fibrosis is unclear [15].

In this study, we aimed to decipher the metabolic consequences of exposing cultured human bronchial epithelial cells (HBECs) to hyperglycemia‐like glucose concentrations and investigated their potential to activate fibroblasts.

## Materials and methods

### Cell culture

#### HBEC cell line

Immortalized human bronchial epithelial cells (HBEC3‐KT) were purchased from ATCC (Manassas, VA, USA; catalog number: CRL‐4051). HBECs were cultured in Airway Epithelial Cell Growth Medium (PromoCell, Heidelberg, Germany) supplemented with 0.004 mL·mL^−1^ bovine pituitary extract, 10 ng·mL^−1^ epidermal growth factor, 5 μg·mL^−1^ insulin, 0.5 μg·mL^−1^ hydrocortisone, 0.5 μg·mL^−1^ epinephrine, 6.7 ng·mL^−1^ triiodo‐l‐thyronine, 10 μg·mL^−1^ recombinant human transferrin, 0.1 ng·mL^−1^ retinoic acid, 10 000 units·mL^−1^ penicillin, and 10 000 μg·mL^−1^ streptomycin. HBEC cultures were maintained in a humidified atmosphere with 5% CO_2_ at 37 °C. HBECs were cultured with low glucose concentration (5 mm) or high glucose concentration (12.5 mm) for 24 or 72 h, with a change to fresh media every 24 h.

#### Fibroblasts

Primary adult human lung fibroblasts were purchased from Lonza (Morristown, NJ, USA; Catalog number: CC‐2512). Fibroblasts were cultured in Fibroblast Growth Medium supplemented with 0.5 mL recombinant human insulin, 0.5 mL human fibroblast growth factor (hFGF‐B), 0.5 mL GA‐1000 (gentamicin and amphotericin B), 10 mL fetal bovine serum (FBS), 10 units·L^−1^ penicillin, and 10 mg·mL^−1^ streptomycin. Fibroblast were cultured in wells coated with 4 μg·mL^−1^ of collagen I in an incubator with 5% CO_2_ at 37 °C.

### Quantitative real‐time PCR

RNA was extracted from cells using the Direct‐zol RNA miniprep kit (Zymo Research, Irvine, CA, USA) as described in the manufacturer's protocol. First‐strand complementary DNA (cDNA) was synthesized from total RNA using High‐Capacity cDNA Reverse Transcription Kit (Applied Biosystems, Pittsburgh, PA, USA) at 25 °C for 10 min, 37 °C for 120 min, 85 °C for 5 min, followed by a cooling step at 4 °C. qRT‐PCR was performed using SYBR Green Master Mix (Applied Biosystems) with the following thermocycling program: (95 °C for 5 min, 95 °C for 15 s, 55 °C for 1 min) × 40 times, followed by 65 to 95 °C (0.5 °C increment) for 5 s. *HPRT1* was used as the housekeeping gene. Primer sequences are provided in Table 1. qPCR results are represented as relative quantification to the 5 mm glucose‐treated cells.

**Table 1:** Sequence of primers used in the study.

Gene name	Forward sequence	Reverse sequence
ACTA2	TGATTAAGGTGGAGGAGTAA	TGATGATGATGATGATGATGAT
CCN2	TTCAGTAGCACAAGTTAT	TTCAGTAGCACAAGTTAT
CCND1	GGCGGAGGAGAACAAACAGA	TGTGAGGCGGTAGTAGGACA
COL1A1	CACCAATCACCTGCGTACAG	CCAGTTCTTGGTCTCGTCAC
COL1A2	TGCCTAGCAACATGCCAATC	CTTTCTCCAGATGGTCCTCT
HK1	ATGGCTGCGGTTGTGGATAA	GATCCCGGACTCTTAGCTGC
HK2	TGGACAACGCAGTGAGACTC	ACTCCTGGGCTCATGATCCT
HPRT1	GACAGGACTGAACGTCTTGC	GCACACAGAGGGCTACAATG
Ki67	CACCTAAGGAAGAGGCCCAA	GGTGGAGATTTGCAGGCTA
P16	TGAGCTTTGGTTCTGCCATT	AGCTGTCGACTTCATGACAAG
P21	GAGACTAAGGCAGAAGATGTAGAG	GCAGACCAGCATGACAGAT
P53	CTGGCATTTGCACCTACCTC	TAACCAGCTGCCCAACTGTA
PCNA	AGCCGAAACCAGCTAGACTT	ACCGCTGGAGCTAATATCCC
PDK1	GAGGGTTACGGGACAGATGC	TTTCCGATGTCCCCAGTGTG
PDK2	TCTCCATCCGCATGCTCATC	CACCTCAGAGACGTTGCAGT
SLC2A1	ACTCATGACCATCGCGCTAG	GGACCCTGGCTGAAGAGTTC
SLC2A10	TGCCCTCAACTATGCACTGG	CTGCCCTGTTAGTTGCTGGA

### Western blot for protein detection

Protein concentrations in cell samples homogenized in RIPA buffer were determined using Pierce BCA Protein Assay Kit (Thermo Fisher, Carlsbad, CA, USA). Laemli sample buffer was added to equal protein amounts of homogenized cell samples and incubated at 70 °C for 5 min. Proteins were then separated by SDS/PAGE and transferred onto nitrocellulose membranes by semi‐dry transfer. Membranes were blocked with 2% bovine serum albumin (BSA) in tris‐buffered saline with Tween 20 (TBST) for 1 h on an orbital shaker, incubated with primary antibody (1 : 1000) in 2% BSA at 4 °C overnight, washed three times with TBST, incubated with secondary antibody (1 : 5000) in 2% BSA at room temperature for 1 h, and washed three times with TBST, all with gentle shaking. Membranes were then developed using SuperSignal West Pico or Femto PLUS Chemiluminescent Substrates (Thermo Fisher). Antibody details are provided in Table 2.

**Table 2:** Antibodies used in this study.

Target protein	Antibody details
p‐PDHA1	Recombinant, monoclonal. AbCam cat# ab177461: Anti‐PDHA1 (phospho S293) antibody [EPR12200]
PDHA1	Recombinant, monoclonal. AbCam cat#ab168379: Anti‐PDHA1 antibody [EPR11098]
α‐SMA	Recombinant, monoclonal. Cell Signaling Technology cat# 19245: α‐Smooth Muscle Actin (D4K9N) XP® Rabbit mAb
COL1A1	Recombinant, monoclonal. Cell Signaling Technology cat# 91144: COL1A1 (E8I9Z) Rabbit mAb
β‐tubulin	Recombinant, monoclonal. Cell Signaling Technology cat# 2128: β‐Tubulin (9F3) Rabbit mAb
p‐16	Recombinant, monoclonal. Cell Signaling Technology cat# 80772: p16 INK4A (D7C1M) Rabbit mAb
p‐21	Recombinant, monoclonal. Cell Signaling Technology cat# 2947: p21 Waf1/Cip1 (12D1) Rabbit mAb
Rabbit IgG	Polyclonal, Cell Signaling Technology cat# 7074: Anti‐rabbit IgG, HRP‐linked Antibody

### Lactate dehydrogenase (LDH) assay

The assay was performed as described in the manufacturer's protocol (Lactate Dehydrogenase Assay Kit, Abcam, Waltham, MA, USA). Cells were washed with cold 1× phosphate‐buffered saline (PBS), scraped, and lysed in 300 μL of cold assay buffer by sonication. Lysed cells were centrifuged at 10 000 ***g*** for 15 min at 4 °C, and the supernatant was collected for the assay. Conditioned media were collected and directly used for the assay. LDH activity was normalized to protein concentration in the cell lysates or in the media.

### α‐Ketoglutarate concentration

The assay was performed as described in the manufacturer's protocol (α‐Ketoglutarate Assay Kit, Cell Biolabs, San Diego, CA, USA). Briefly, cells were washed with PBS and lysed in 300 μL of PBS by sonication. Lysed cells were centrifuged at 10 000 ***g*** for 10 min at 4 °C, and the supernatant was used for the assay.

### Triglyceride assay

Lipids were extracted from HBEC lysates using the Folch method [16]. Triglyceride quantification was performed using the manufacturer's protocol (L‐Type Triglyceride M, Wako, Richmond, VA, USA). The triglyceride amount was normalized to the protein amount in cell lysates.

### Glucose‐6‐phosphate dehydrogenase activity

The assay was performed as described in the manufacturer's protocol (Glucose‐6‐Phosphate Dehydrogenase Activity Assay Kit, Cell Biolabs). Briefly, cells were washed with cold 1× PBS and lysed in 300 μL of cold 1× Lysis Buffer by incubating on ice for 10 min. Lysed cells were centrifuged at 10 000 ***g*** for 10 min at 4 °C, and the supernatant was collected for the assay.

### Glycogen quantification

The assay was performed as described in the manufacturer's protocol (Glycogen Assay Kit, Cell Biolabs). Cells were washed with PBS and lysed in 300 μL of PBS by sonication. Lysed cells were centrifuged at 10 000 ***g*** for 10 min at 4 °C, and the supernatant was used for the assay.

### Cell proliferation assay

Cell proliferation profiles of HBECs were determined by performing continuous live cell assay using the BioTek Cytation 5 and Agilent Bio Tek BioSpa (Santa Clara, CA, USA), a fully automated cell counting system. HBECs were incubated in BioSpa at 37 °C, 5% CO_2_ and 90% humidity for 72 h. Brightfield images were taken every 4 h, and media were changed every 24 h. Cell counts were calculated from the images at each time point using the biotek gen5 software.

### Oxidative DNA damage analysis

DNA was extracted from HBECs as described in the manufacturer's protocol (Quick‐DNA Miniprep Kit, Zymo Research). 8‐Hydroxydeoxyguanosine (8‐OHdG), a ubiquitous marker of oxidative DNA damage, was quantified using the OxiSelect Oxidative DNA Damage ELISA kit (Cell Biolabs). The assay was performed as described in the manufacturer's protocol.

### Oxidative protein damage analysis

Protein carbonyl assay was performed on HBECs as outlined in the manufacturer's protocol (OxiSelect Protein Carbonyl ELISA kit, Cell Biolabs).

### β‐Galactosidase staining

The assay was performed as instructed in the manufacturer's protocol (Cell Signaling, Danvers, MA, USA).

### Antibody array for the detection of proteins in conditioned media

Media conditioned by HBECs cultured in low or high glucose for 72 h were used. The assay was performed as described in the manufacturer's protocol (RayBio C‐Series Human Cytokine Antibody Array C2000, RayBiotech, Peachtree Corners, GA, USA) using media pooled from six individual wells per condition.

### Oxygen consumption rate (OCR) measurement

OCR of HBEC cultures was measured real‐time using the Lucid Scientific Resipher device. HBECs were cultured on a 96‐well plate with low or high glucose media for 72 h with the Resipher sensing lid, which continuously records oxygen levels in the media. Resipher web application was used to analyze the data, and OCR was presented as fmol·mm^−2^·s^−1^. The media were renewed every 24 h during the experiment.

### Statistical analysis

The majority of the data are expressed as dot plots with the means ± SD highlighted. A comparison of groups was performed by Student's *t*‐test (two‐tailed). Experiments with more than two groups were analyzed by two‐way ANOVA with Bonferroni posttests analysis. *P* values for significance were set at 0.05 All analyses were performed using the graphpad prism Software (Version 9; Boston, MA, USA).

## Results

### Extracellular glucose availability determines intracellular glucose metabolism in HBECs

**Fig. 1:**
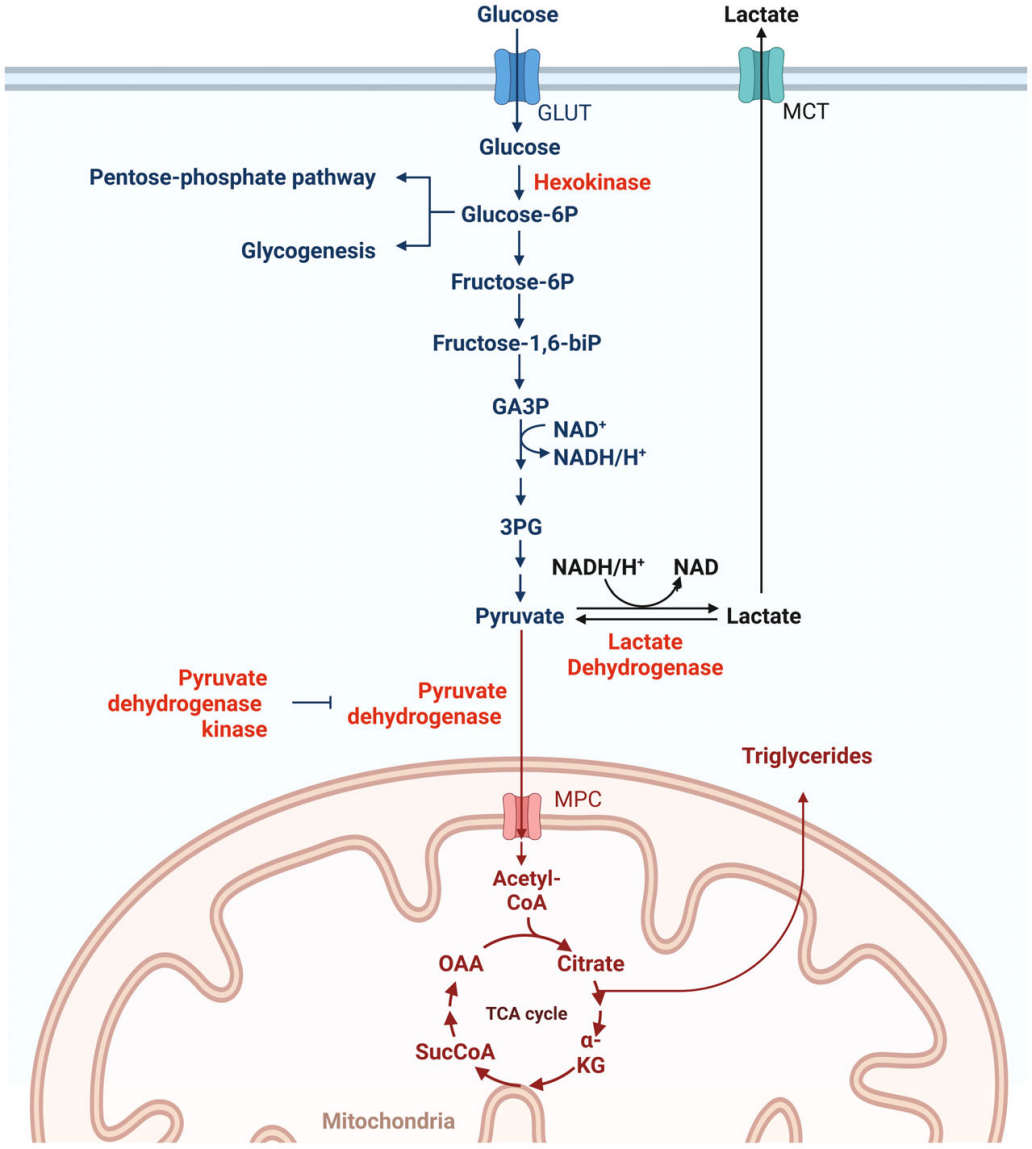
Schematic of major intracellular metabolic pathways using glucose as substrate. Once the glucose enters the cell through a glucose transporter (GLUT), it is phosphorylated to glucose‐6‐phosphate (G6P) by hexokinase. G6P can enter the pentose phosphate pathway (PPP), the glycogen synthesis pathway or be further metabolized in the glycolysis pathway to generate pyruvate. Pyruvate can be metabolized to acetyl‐CoA by pyruvate dehydrogenase in the mitochondria and then enter the tricarboxylic acid (TCA) cycle or exit the mitochondria to be used for fatty acid and triglyceride synthesis. Pyruvate can also be anaerobically metabolized into lactate by cytosolic lactate dehydrogenase (LDH), followed by secretion to the extracellular environment through a monocarboxylate transporter (MCT). Other abbreviations in the figure: 3PG, 3‐phosphyglycerate; GA3P, glyceraldehyde 3‐phospate; NAD, Nicotinamide adenine dinucleotide; OAA, oxaloacetate; α‐KG, alpha ketoglutarate. Figure created with Biorender.com.

**Fig. 2:**
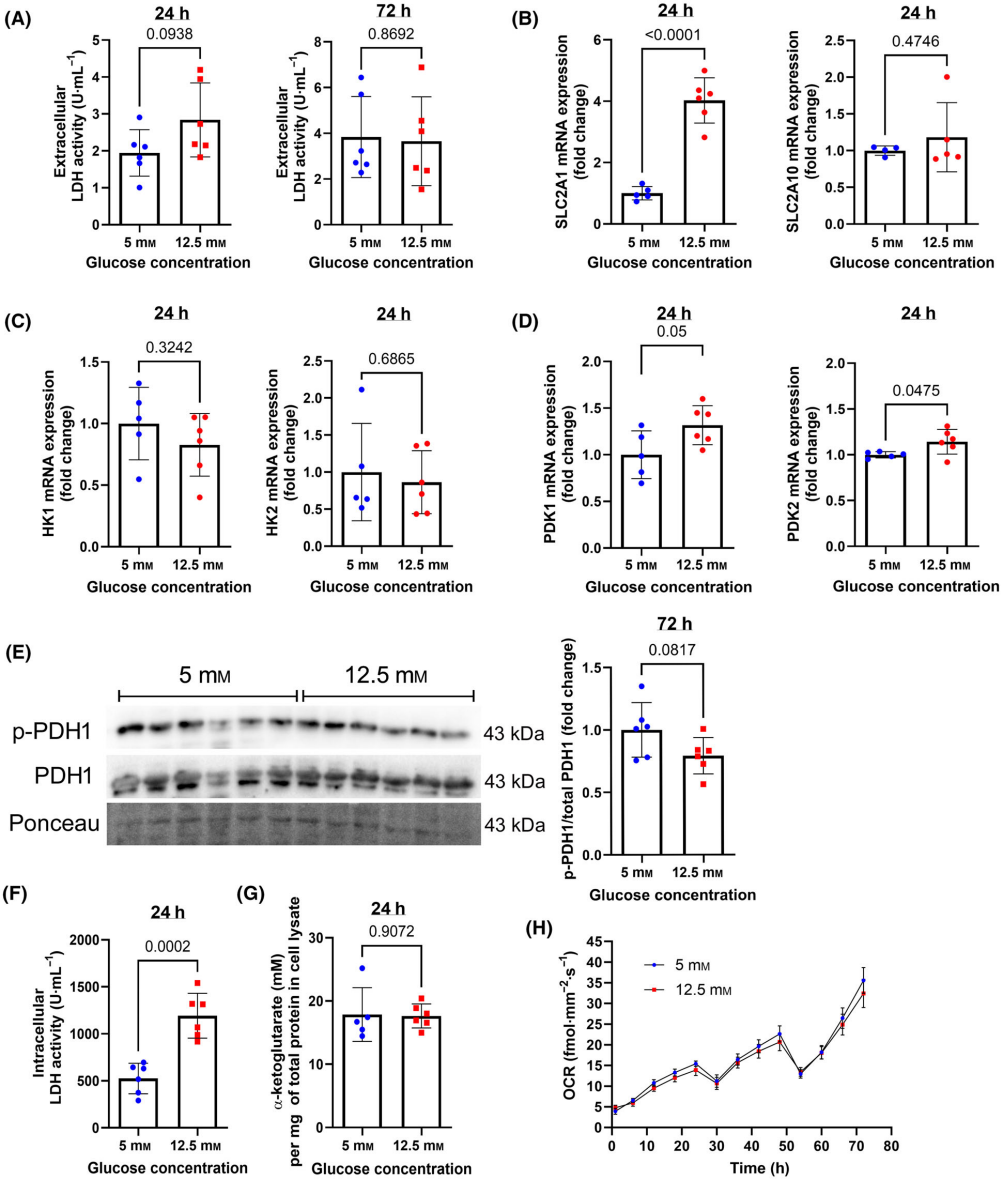
Effects of low and high concentration of glucose on glycolysis, TCA cycle and cellular respiration in HBECs. (A) Extracellular LDH activity was measured at 24 and 72 h of culture for assessment of toxicity. (B) qPCR assessment of mRNA expression of *SLC2A1* and *SLC2A10*, *HK1* and *HK2* (C), *PDK1* and *PDK2* (D). (E) Western blot phosphorylated and total PDH1, Ponceau staining of the membrane region, and densitometry quantification of phospho‐PDH1/total PDH1. (F) Quantification of intracellular LDH and α‐ketoglutarate (G) activity. (H) Oxygen consumption rate (OCR) measured in real‐time for 72 h. The dips in the curve coincide with media change timepoints. AUC, area under the curve. Data were analyzed by unpaired *t*‐test. *N* = 5 to 6. Mean ± SD and *P* values are shown in each graph.

To investigate how metabolic pathways (Fig. 1) respond to high glucose concentration in the bronchial epithelium, an immortalized HBE cell line was cultured in media supplemented with glucose at concentrations 5 or 12.5 mm for 24 or 72 h. These glucose levels are considered still physiological in a hyperglycemic context, and below the levels that would trigger blood hyperosmolarity and associated additional cell responses and complications [17]. LDH activity in the media was measured to discard potential cytotoxic effects of high glucose concentration. No change in cytotoxicity was observed after 24 and 72 h of high glucose exposure, and hence, those concentrations were used for the study (Fig. 2A). Importantly, glucose levels *in vivo* are not constant and vary with fasting or postprandial conditions. In our cell cultures, media were refreshed every 24 h to replenish the original glucose concentration. As cells grew, they consumed glucose in each 24‐h period as follows: In the cultures containing 5 mm glucose, the concentration descended to 1.1 ± 0.07 mm after 24 h, to 1.1 ± 0.04 mm after 48 h, and to 0.3 ± 0.07 mm after 72 h since the start point. In the cultures that contained 12.5 mm glucose, the concentration descended to 2.1 ± 0.04 mm after 24 h, to 1.9 ± 0.05 mm after 48 h, and to 2.4 ± 0.04 mm after 72 h. Unless otherwise indicated, all data were collected at 24 h of exposure.

Although there is notably scarce knowledge on the use of glucose by pulmonary cells, it is known that imported glucose enters the same intracellular metabolic pathways described for all eukaryotic cells. GLUT1, GLUT2, and GLUT10, encoded by *SLC2A1*, *SLC2A2*, and *SLC2A10*, respectively, are expressed in HBECs [6]. HBECs exposed to high glucose concentration showed a fourfold increase in the expression of *SLC2A1* compared with HBECs cultured in low glucose (Fig. 2B). In contrast, *SLC2A10* expression was not changed in response to high glucose, and *SLC2A2* was not detected in our cultures. Gene expression of hexokinases I and II (*HKI* and *HKII*), which catalyze glucose phosphorylation after it enters the cells, was not altered in response to high glucose exposure (Fig. 2C).

Next, the use of glucose by HBECs for cellular energy demands was assessed in the two different glucose conditions. Glycolysis generates pyruvate that can be used for aerobic mitochondrial oxidation or anaerobic synthesis of lactate (Fig. 1). Gene expression of pulmonary isoforms 1 and 2 of pyruvate dehydrogenase kinase (PDK 1 and 2) [18] was significantly upregulated in HBECs in response to extracellular high glucose concentration (Fig. 2D). PDK catalyzes an inactivating phosphorylation of pyruvate dehydrogenase (PDH; Fig. 1). Western blot detection showed no changes in the ratio of phosphorylated to total PDHA1 between cells exposed to 5 or 12.5 mm glucose (Fig. 2E), suggesting that the additionally available glucose was not entering into the mitochondria. Intracellular LDH activity increased by 2.3‐fold in HBECs exposed to high glucose concentration (Fig. 2F), suggesting that glycolysis‐resulting pyruvate was anaerobically oxidized to lactate, even in the presence of oxygen.

Consistently, mitochondrial pathways showed no differences between the cells exposed to the two different concentrations of extracellular glucose. The concentration of α‐ketoglutarate, an intermediate of the tricarboxylic acid cycle (TCA), and OCR were similar for both conditions (Fig. 2G,H).

**Fig. 3:**
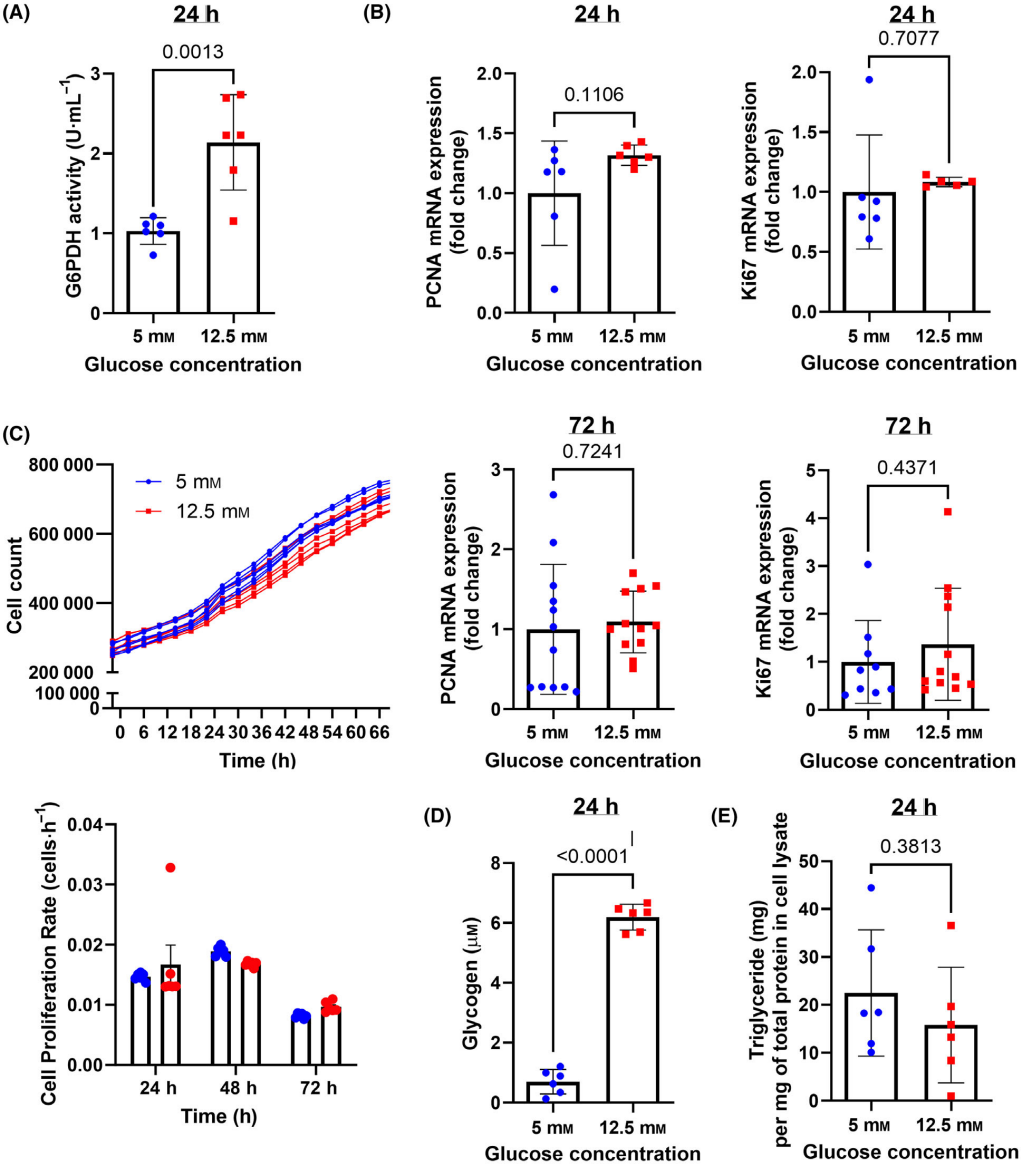
Effect of low and high concentration of glucose on non‐mitochondrial metabolic pathways of glucose and cell proliferation in HBECs. (A) Quantification of G6PDH activity in HBECs cultured in low or high glucose media for 24 h. (B) Gene expression of proliferation markers *PCNA* and *Ki67* measured by qRT‐PCR. (C) HBECs were counted in culture wells every 4 h for 72 h and proliferation rate was calculated. *PCNA* and *Ki67* were measured by qPCR at the end of the experiment at 72 h (right). (D) Intracellular amounts of glycogen and (E) triglycerides at 24 h of culture. Data were analyzed by unpaired *t*‐test. *N* = 5 to 12. Mean ± SD and *P* values are shown in each graph.

Besides pyruvate, intracellular glucose‐6‐phosphate (G6P) generates ribose‐5‐phosphate and nicotinamide adenine dinucleotide phosphate (NADPH) through the pentose phosphate pathway (PPP). The activity of glucose‐6‐phosphate dehydrogenase (G6PDH), the rate‐limiting enzyme of the PPP, increased by twofold in high glucose‐exposed HBECS (Fig. 3A). Since the pyridine nucleotides and NADPH resulting from the PPP can be used for membrane and DNA synthesis during cell proliferation, the expression of proliferation markers was measured. There were no changes in the gene expression of the markers proliferating cell nuclear antigen (PCNA) and Ki67 in HBECs exposed to high glucose for 24 or 72 h (Fig. 3B). Consistently, automated cell counting did not detect any differences in cell proliferation rates between normal and high glucose‐exposed HBECs (Fig. 3C).

Finally, other intracellular fates of glucose include energy storage as triglycerides via *de novo* lipogenesis and glycogen via glycogenesis. There was an 8.8‐fold increase in the amount of glycogen present in HBECs cultured in high glucose media, while no difference in intracellular triglyceride levels was observed between the two conditions (Fig. 3D,E).

Therefore, when extracellular glucose is available in high concentration, HBECs do not significantly change their mitochondrial metabolism or proliferation rate, and instead store glucose as glycogen.

### Exposure to high glucose concentration did not induce oxidative damage or senescence in HBECs

**Fig. 4:**
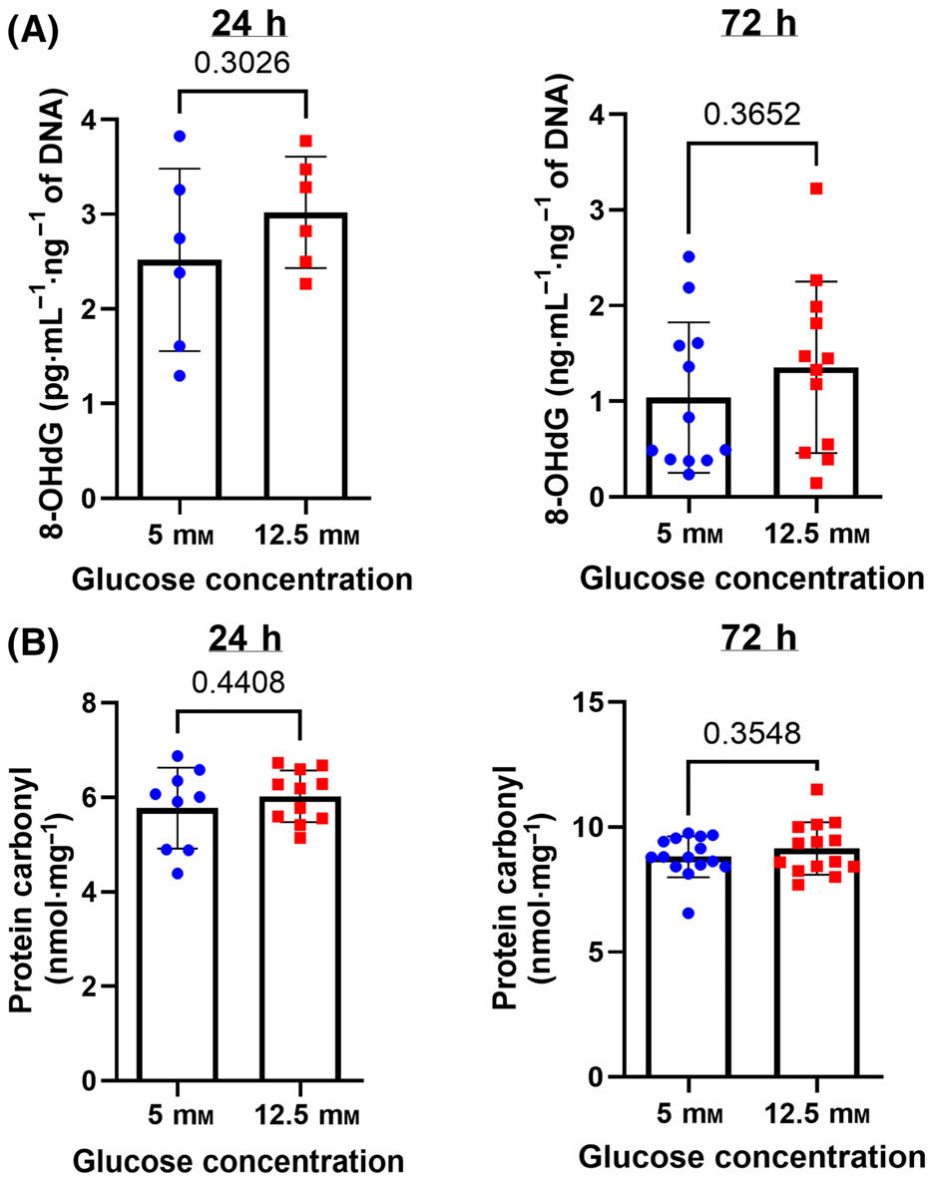
Evaluation of oxidative DNA and protein damage in HBECs cultured with low or high glucose concentration measured at 24 and 72 h. (A) 8‐OhdG as an indicator of oxidative DNA damage and (B) protein carbonyl as an indicator of oxidative protein damage were measured in HBECs cultured in low and high glucose media. Data were analyzed by unpaired *t*‐test. *N* = 6–12. Mean ± SD and *P* values are shown in each graph.

Prolonged exposures to high concentrations of extracellular glucose are associated with ROS production and oxidative damage [19]. However, oxidative damage was not observed in our experimental setting. Oxidative DNA damage did not differ between HBECs cultured in low or high glucose media for 24 or 72 h (Fig. 4A). Similarly, protein carbonyls, the long‐term product of protein oxidation by ROS, were also unchanged in HBECs exposed to high glucose for 24 and 72 h (Fig. 4B).

**Fig. 5:**
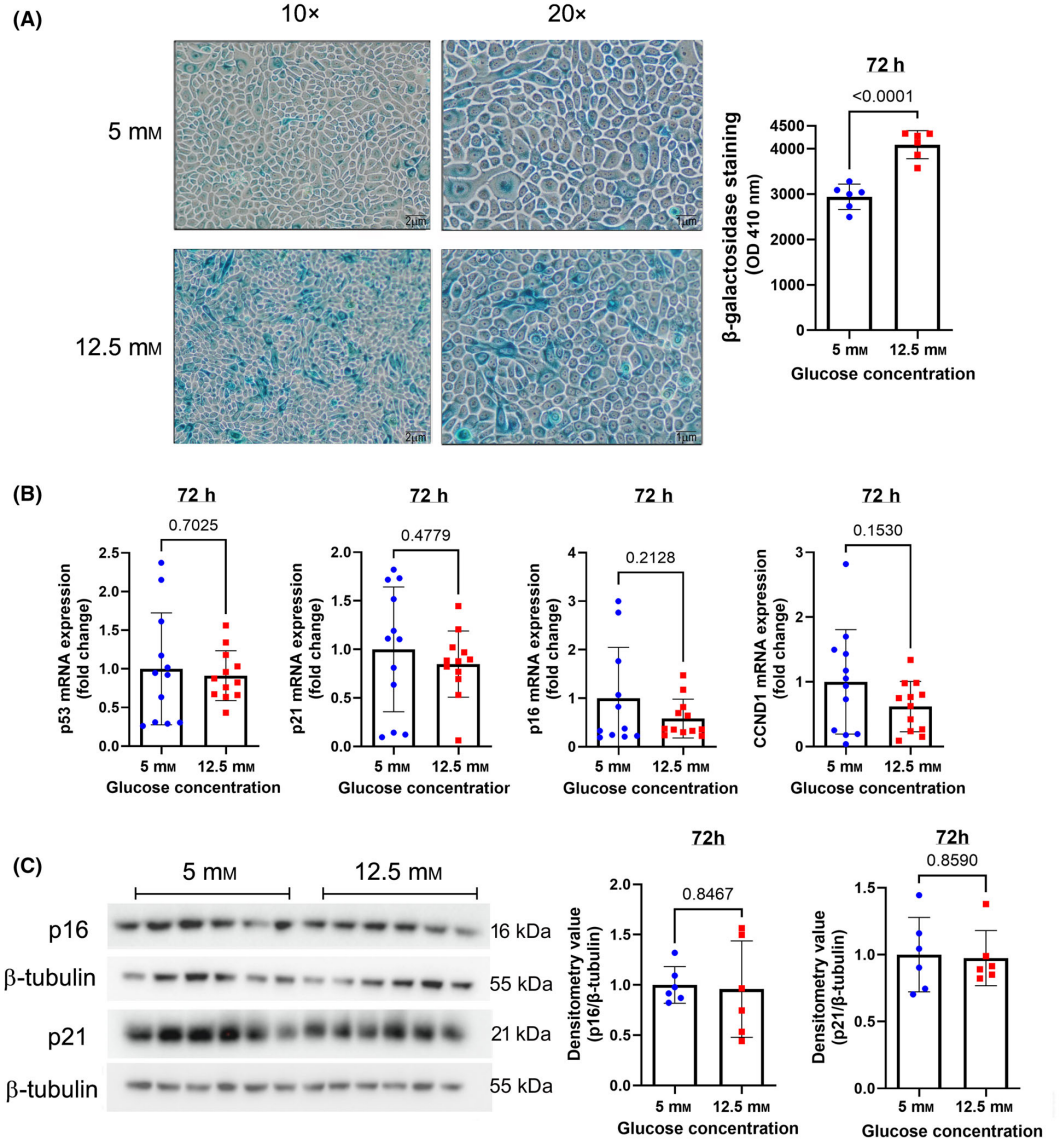
Evaluation of cellular senescence in HBECs cultured with low or high glucose concentration. (A) Representative images of β‐galactosidase staining in HBECs cultured in low and high glucose media for 72 h (10× and 20× magnification). (B) Gene expression of p53, p21, p16, and CCND1 measured by qRT‐PCR. (C) western blot for p16 and p21 in HBECs cultured in low and high glucose media (left) and quantification of measured densitometry values (right). Data were analyzed by unpaired *t*‐test. *N* = 6 to 12. Mean ± SD and *P* values are shown in each graph.

Senescence is another deleterious effect of long‐term exposure to high concentrations of glucose in human pulmonary epithelial cells [10, 11]. β‐Galactosidase staining, a hallmark of senescence, was increased in HBECs exposed to high glucose conditions for 72 h (Fig. 5A). However, mRNA expression levels of senescence markers involved in cell cycle regulation *TP53* (encoding p53), *CDKN1A* (encoding p21), *CDKN2A* (encoding p16), and cyclin D1 did not show differences between HBECs cultured in low and high glucose media for 72 h (Fig. 5B). In line with this, no difference in protein expression of p16 and p21 was observed by western blot between the two conditions (Fig. 5C), suggesting that HBECs do not become senescent under these conditions.

### HBECs cultured in high glucose conditions induced fibroblast activation

**Fig. 6:**
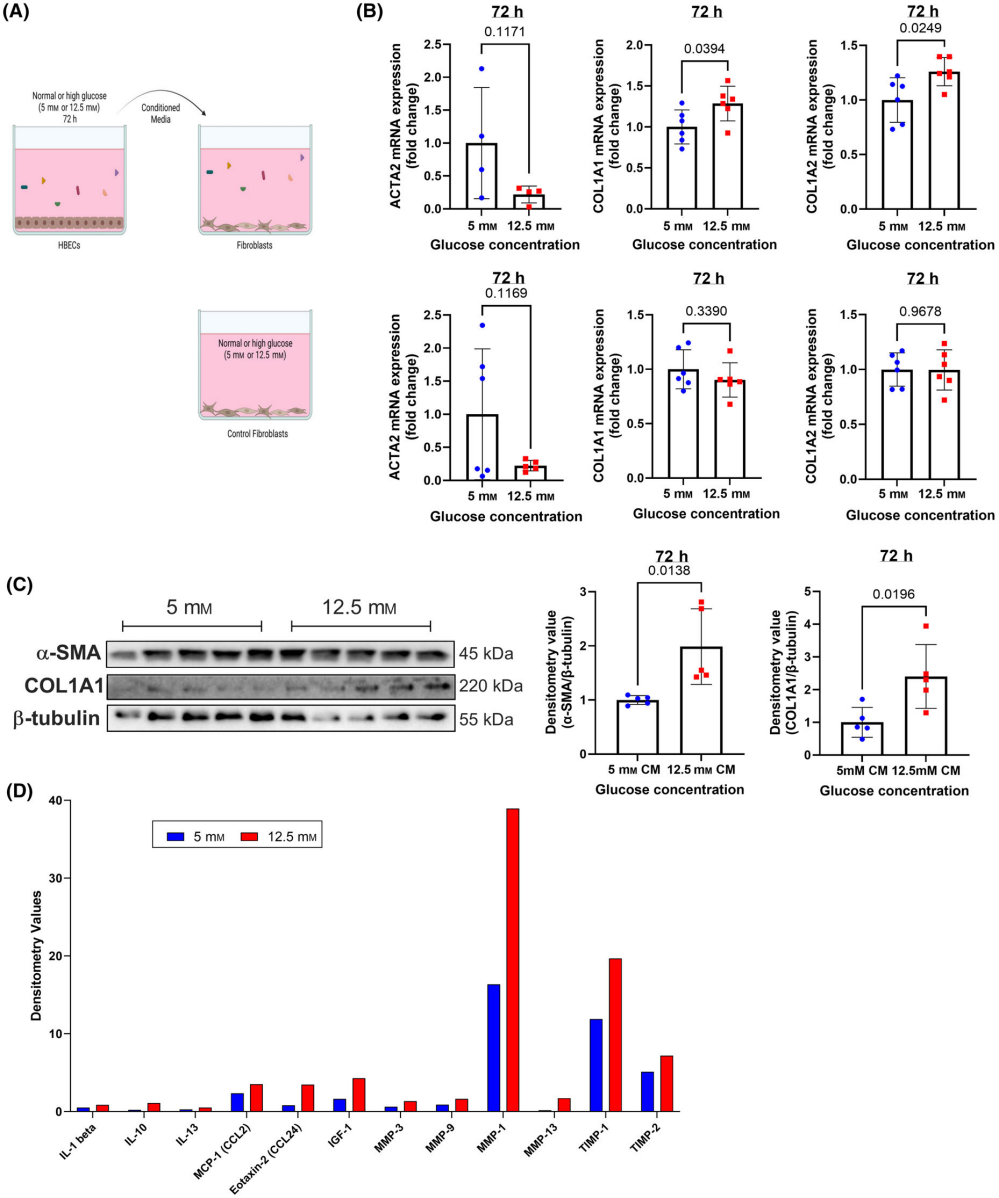
Assessment of fibroblast activation. (A) Schematic showing the experimental design for the generation of conditioned media and exposure to fibroblasts, using fresh media as the control condition. (B) qRT‐PCR for gene expression of *ACTA2*, *COL1A1* and *COL1A2* in human lung fibroblasts. The top panels show fibroblasts cultured in in HBECs‐conditioned media, with low or high glucose and the bottom panels show fibroblasts cultured with fresh media containing low or high glucose. (C) Western blot of α‐SMA and COL1A1 in human lung fibroblasts exposed to HBEC‐conditioned media (left) and densitometry values normalized to β‐tubulin (right). (D) Proinflammatory and profibrotic mediators released into the media by HBECs exposed to low or high glucose detected by protein array using pooled media from 6 wells per group. Densitometry values for each marker were measured and normalized to the positive controls within the array. *N* = 4 to 10. Mean ± SD and *P* values are shown in each graph. CM, conditioned media.

HBECs are exposed to high concentrations of glucose in patients with hyperglycemia, and airway fibrotic remodeling is associated with T1D [12]. Whether epithelial cells exposed to high glucose concentration can activate collagen synthesis by adjacent fibroblasts is unclear. We exposed primary human lung fibroblasts to media previously conditioned by HBECs at low or high glucose conditions for 72 h (Fig. 6A). Fresh media with the two glucose concentrations was used to account for the potential direct effects of glucose on fibroblast activation.

Gene expression of activation markers *COL1A1* and *COL1A2* increased by 1.3‐fold in fibroblasts cultured with media conditioned by HBECs exposed to high glucose (Fig. 6B top panels), while there were no differences between fibroblasts exposed to 5 or 12 mm glucose‐containing fresh media (Fig. 6B bottom panels). This increase was also observed at the protein level too for COL1A1 (Fig. 6C). Further indicating fibroblast activation, protein expression of alpha smooth muscle actin (α‐SMA) also increased by twofold, despite no change in *ACTA2* gene expression.

A protein array was used to analyze the HBECs‐conditioned media to detect potential mediators that could activate fibroblasts. High glucose increased the media concentration of interleukins IL‐1β, IL‐10, and IL‐13, as well as CC chemokine ligands (CCLs), CCL2 and CCL24 (Fig. 6D). Media conditioned by high glucose‐exposed HBECs also showed higher concentrations of insulin growth factor 1 (IGF‐1), metalloproteinases (MMPs), MMP‐1, ‐3, ‐9 and ‐14 and tissue inhibitors of MMPs (TIMPs), TIMP‐1 and ‐2. Those mediators were previously described as contributing to pro‐fibrotic effects through different mechanisms [20, 21, 22, 23, 24]. These data suggest that, in response to high glucose exposure, HBECs release profibrotic mediators that increase adjacent fibroblast collagen synthesis.

## Discussion

This study aimed to identify the metabolic pathways used by HBEC in response to different concentrations of extracellular glucose availability. Our data showed that HBECs exposed to high concentrations of glucose, equivalent to plasma levels encountered in diabetic conditions, metabolize the excess substrate through the PPP and lactate synthesis, and also store it as glycogen.

Exposure to high concentrations of glucose upregulated GLUT1 expression at the mRNA and protein levels in the retina and kidney [25, 26]. In this study, HBECS showed increased mRNA expression of *SLC2A1*, the gene encoding glucose transporter GLUT1, suggesting that GLUT1 was likely the primary transporter. GLUT1 is an insulin‐insensitive glucose transporter [27, 28] and is coupled with hexokinase I phosphorylation of glucose [29], ensuring glucose entry favoring a concentration gradient. Glucose is rapidly metabolized via hexokinase‐dependent pathways in response to high glucose in HBECs without change in hexokinase protein or mRNA abundance [7]. Increased glucose availability as energy substrate could trigger increased flux through several metabolic pathways downstream uptake, but not all pathways were equally impacted in HBECS (below).

PDK modulation of the PDH complex and thus the entry of glycolysis‐derived pyruvate into the TCA cycle positions this enzyme as a switch between glycolytic and lipolytic metabolism [30]. Our oxygen consumption, PDH phosphorylation assessment, and triglyceride measurement data show that HBECs did not increase mitochondrial metabolism and instead generated increased amounts of lactate through LDH activity, averting a substitution of fatty acids by glucose as an energy substrate. Accumulation of triglycerides in the bronchial epithelium due to altered lipid metabolism is associated with increased airway resistance and impaired bronchiolar regeneration in mice [31]. However, in our study, excess glucose available was not redirected for potentially deleterious lipogenesis and triglyceride synthesis, but it was stored as glycogen instead. To our knowledge, accumulation of glycogen in the lung is not a common occurrence. Accumulation of glycogen in developing fetal lungs in mammals is suggested to be involved in later phospholipid synthesis by alveolar but not airway epithelium [32, 33], and increased glycogen level is associated with lung carcinoma [34, 35]. However, the main function of glycogen in epithelial cells may be to support the synthesis of the glycocalyx, which is composed of carbohydrates such as heparan sulfate and proteoglycans [36]. The roles of glycocalyx synthesized by epithelial and endothelial cells during lung disease is an intense area of research [37].

Cancer and other proliferating cells often show increased LDH activity to support the energy demands of fast cell proliferation [38], as well as increased glucose flux into the PPP to meet the demand for nucleotide and amino acid biosynthesis. HBECs did not show increased proliferation rates in this study, which indirectly suggests a limited potential for the use of glucose as a substrate to support reepithelization after injury. Increased flux through the PPP would result in higher availability of reducing power in the form of NADPH, which can be used for the generation of reduced glutathione to counteract oxidative stress. Consistently, HBECs showed no signs of oxidative stress in response to high glucose concentration, despite oxidative stress being often observed in the lungs of T1D animal models and IPF patients [39, 40].

Increased lactate production was observed in lung tissues and alveolar type 2 cells isolated from patients with pulmonary fibrosis, and lactate successfully induced differentiation of primary human lung fibroblast into myofibroblasts [41]. In a different study, HBECs exposed to 15 mm glucose also demonstrated a metabolic shift toward increased intracellular lactate production and enhanced secretion of lactate into the ASL [42]. In our study, HBECs exposed to high glucose concentration increased LDH activity and secreted higher levels of proinflammatory and proteolytic markers into the media. Amongst them, IL‐1β, IL‐10, and IL‐13 are associated with pulmonary fibrosis [20, 43, 44]. CCL2, CCL24, and IGF‐1 can activate fibroblasts [21, 22], and MMPs and TIMPs are involved in ECM remodeling and fibrotic responses [23, 24]. Consistently, primary human lung fibroblasts exposed to media conditioned by high glucose‐exposed HBECs increased the gene expression of *COL1A1* and *COL1A2* and protein expression of α‐SMA and COL1A1 in our study. Whether the definite activator of fibroblasts is lactate, any of the cytokines detected in the media, or a combination of several factors would require extensive research. However, these data show that epithelial cells can contribute to fibroblast activation in diabetes‐associated pulmonary fibrosis.

Similarly, the mechanisms linking increased glucose availability with the secretion of proinflammatory and profibrotic mediators could be heterogeneous. We did not observe oxidative stress or senescence in this study. β‐Galactosidase staining, which was increased in our study, can also be elevated in non‐senescent cells [45]. Activity of lysosomal β‐galactosidase at pH 6 has been shown to be increased in senescent cells and tumors, and it is commonly used as marker of replicative senescence [46]. However, it is not a specific maker as it may just reflect increased lysosomal activity regardless of other senescent markers. Senescence occurs in cells from patients with autosomal recesive GM1‐gangliosidosis, who lack lysosomal β‐galactosidase protein [47], and increased β‐galactosidase activity can be induced *in vitro* at low passage numbers by exposure to cigarette smoke extract [48]. A limitation of this study is that the HBEC cell line is immortalized through modifications of human telomerase (hTERT), which could interfere with senescence processes [49]. Follow‐up experiments will benefit from using primary cells to overcome this limitation. Indeed, experiments on fully differentiated primary airway epithelial cells cultured at the air‐liquid interface and in combination with adapted techniques to measure bioenergetics data, as in [50], could overcome the limitation of using cell lines that in submerged conditions may not acquire fully differentiated phenotypes.

In conclusion, this study showed that when exposed to glucose concentrations comparable with hyperglycemic conditions, HBECs can store glucose as glycogen and are protected from glucose‐induced oxidative stress, but they activate neighboring fibroblasts for collagen production. The implications are relevant for understanding the metabolic crosstalk of neighboring cells in complicated diseases such as diabetes‐associated pulmonary fibrosis.

## Conflict of interest

The authors declare no conflict of interest.

## Author contributions

SSP was involved in methodology, validation, formal analysis, investigation, writing—original draft, and writing—review and editing. RW was involved in validation, formal analysis, and writing—review and editing. PG was involved in conceptualization, validation, investigation, writing—review and editing, and supervision. IG‐A was involved in conceptualization, methodology, validation, formal analysis, resources, writing‐original draft, writing—review and editing, supervision, project administration, and funding acquisition.

## Data Availability

The data that support the findings of this study are contained in the tables, figures, and text of this article.
